# Eye-tracking for assessing medical image interpretation: A pilot feasibility study comparing novice vs expert cardiologists

**DOI:** 10.1007/s40037-019-0505-6

**Published:** 2019-04-11

**Authors:** Tad T. Brunyé, Brahmajee K. Nallamothu, Joann G. Elmore

**Affiliations:** 1 Center for Applied Brain & Cognitive Sciences, Tufts University, Medford, MA USA; 2 Division of Cardiovascular Diseases, University of Michigan, Ann Arbor, MI USA; 3 Department of Medicine, University of Washington, Seattle, WA USA

**Keywords:** Cardiology, Decision making, Eye tracking, Cognitive psychology

## Abstract

**Introduction:**

As specialized medical professionals such as radiologists, pathologists, and cardiologists gain education and experience, their diagnostic efficiency and accuracy change, and they show altered eye movement patterns during medical image interpretation. Existing research in this area is limited to interpretation of static medical images, such as digitized whole slide biopsies, making it difficult to understand how expertise development might manifest during dynamic image interpretation, such as with angiograms or volumetric scans.

**Methods:**

A two-group (novice, expert) comparative pilot study examined the feasibility and utility of tracking and interpreting eye movement patterns while cardiologists viewed video-based coronary angiograms. A non-invasive eye tracking system recorded cardiologists’ (*n* = 8) visual behaviour while they viewed and diagnosed a series of eight angiogram videos. Analyses assessed frame-by-frame video navigation behaviour, eye fixation behaviour, and resulting diagnostic decision making.

**Results:**

Relative to novices, expert cardiologists demonstrated shorter and less variable video review times, fewer eye fixations and saccadic eye movements, and less time spent paused on individual video frames. Novices showed repeated eye fixations on critical image frames and regions, though these were not predictive of accurate diagnostic decisions.

**Discussion:**

These preliminary results demonstrate interpretive decision errors among novices, suggesting they identify and process critical diagnostic features, but sometimes fail to accurately interpret those features. Results also showcase the feasibility of tracking and understanding eye movements during video-based coronary angiogram interpretation and suggest that eye tracking may be valuable for informing assessments of competency progression during medical education and training.

## What this paper adds

Eye tracking holds potential to allow educators and mentors to monitor and assess student progress in medical disciplines involving medical image interpretation such as radiology and pathology. However, no research has explored whether eye tracking might be useful for assessing the review of dynamic cardiology images. This paper is the first of its kind to consider whether eye tracking might be a valuable tool for identifying and quantifying the development of visual interpretive skills in novice cardiologists interpreting coronary angiograms.

## Introduction

A physician’s expertise in processing visual data is fundamental to accurately interpreting medical images, such as those found in cardiology, pathology, radiology, and dermatology practice [1]. Emerging research at the intersection of medicine, education, and cognitive science has recently revealed reliable differences in visual behaviour during expertise development. For instance, experts tend to move their eyes differently compared with novices, are faster and more accurate in identifying suspicious regions in a visual image, and are less vulnerable to the distracting effects of diagnostically irrelevant visual patterns [2–4]. This research informs curricula and assessment methods for medical education and training, suggesting novel techniques for accelerating novice learning [5] and objectively assessing competency development [6]. However, to date this existing research is restricted to reviewing static images, which is particularly unfortunate given that several medical specialties increasingly involve the review of dynamic visual imagery, such as when reviewing coronary angiograms or volumetric CT scans, or performing diagnostic fluoroscopy or laparoscopy. In these domains, educating and training the visual interpretive process is the linchpin to accurate diagnostic decision making.

Over the course of gaining medical experience, learners develop highly specialized expertise in processing visual data that guides and focuses attention and affords accurate mappings from perceived stimuli to candidate diagnoses. One prominent theory proposes that developing global perceptual strategies allows experts to make fine-grained distinctions between visual stimuli [7–9], with experts encoding broader visual information than novices and quickly developing a relatively holistic representation of an overall configuration [10–12]. Interestingly, much of this holistic processing can be done very quickly and without requiring the expert to fixate his or her eyes on the more global structure [13]. Experts develop specialized skills in visual search, recognizing objects, and making decisions, resulting in higher efficiency in knowing where to look, what to look for, and what it means [14–18]. Eye tracking provides an innovative tool for quantifying and possibly accelerating this expertise development and providing a basis for objective, formative feedback.

### Eye tracking

Many experience-based differences in the visual interpretive process have been revealed through eye tracking [2, 4, 11–13]. Monitoring eye movements is valuable for objectively characterizing the visual search process and in some cases predicting diagnostic outcomes. For the present study, eye movements can be parsed into two meaningful units: fixations and saccades. Fixations describe the momentary pauses of the eyes to foveate a restricted region of space, and saccades describe the rapid, ballistic movement of the eyes between successive fixations [19]. Fixations are characterized by their location in the visual world, and their duration; in general, the more fixations and longer their duration, the more visual attention and interest the viewer has in a region [20]. Saccades are thought to be preceded by an attentional shift to a different location, which results in a saccade to afford foveation of a new goal region [21, 22]. Saccades can reveal how dramatic (usually measured in degrees, or amplitude) the shifts of attention are across a scene. The peak velocity of saccades can help researchers gain insight into varied states of workload and arousal. Specifically, higher peak saccade velocity correlates with greater sympathetic nervous system activation, for instance during states of arousal or uncertainty [23], possibly driven by excitatory inputs to oculomotor neurons from the reticular formation [24, 25].

Tracking the allocation of visual attention over a medical image allows us to dissociate between a failure to view critical scene regions, versus a failure to accurately interpret those regions [26]. Specifically, if a physician fails to fixate on critical regions of a medical image, any diagnostic decision errors that accrue can be sourced to a failure to find critical diagnostic regions. In contrast, if a physician fixates on critical regions but does not arrive at an accurate diagnostic decision, the error can be attributed to the interpretive process. This distinction is critically important for identifying possible sources of error and how they change as a function of expertise development, and informing the development of tailored student assessments and training curricula. For example, eye tracking can exemplify expert eye movements to medical students, allowing them to learn viewing strategies for reviewing complex slides [5]. Eye tracking also holds potential for evaluating student competency progression, objectively assessing skill development and providing formative feedback [6, 27].

Earlier research using eye tracking to understand expertise-related differences in visual search among medical professions is restricted to examining static images due to the complexity of tracking and interpreting eye movements over moving (dynamic) scenes [28, 29]. This is because not only are the head and eyes constantly moving relative to the computer monitor, but also relative to a moving scene with constantly changing stimulus locations. For instance, for a cardiologist reviewing an angiogram, a region of diagnostic importance may move across a scene as tool angulation or position changes, necessitating a tedious frame-by-frame coupling of region location and eye location over time. For this reason, most studies examining eye movements with medical images artificially restrict zooming and panning behaviour, simplifying analysis but also possibly reducing relevance to behaviour elicited during routine clinical practice [26, 30, 31].

To increase the efficiency of interpreting eye movements over dynamic scenes, researchers can temporally couple eye tracking with logged interface behaviour to relate eye position to specific video frames [32–34]. To prioritize video frames, a behaviour analysis can assist in inferring a viewer’s interest in certain video frames: using the *ShotRank *technique, the frequency and duration of viewing video frames indicates interest in the information available on those frames [35–37]. Specifically, repeated interest in certain video frames (e.g., pausing, reviewing) can provide important information regarding the spatiotemporal distribution of critical diagnostic regions throughout a video. The *ShotRank *technique was applied herein to prioritize analysis of video time frames and associated eye movements. Some automated image processing and dynamic region of interest tracking techniques are also becoming available in eye tracking software packages [38–40], but remain heavily reliant on researchers manually defining and correcting regions over the course of the video.

Over three million angiograms are performed each year in the United States and European Union [41, 42], with diagnosis and treatment decisions relying on a cardiologist’s interpretation of dynamic angiogram images, yet the underlying interpretive process has not been adequately studied. Thus, this pilot study provided a first examination of the behaviours and eye movements characterizing cardiologists’ interpretation of coronary angiograms, with implications for the development of next-generation educational and training curricula and assessment methods.

## Methods

Ethics approval was granted by the University of Michigan Institutional Review Board (HUM120284), participants provided written informed consent and all research was carried out in accordance with the Declaration of Helsinki.

### Participant sample

Eight physicians with a range of experience in interventional cardiology were recruited, including six residents and two faculty experts.

### Materials & Equipment

#### Image test set

A test set of eight digitized (DICOM format) videos of a single patient’s coronary angiogram was selected from an anonymized and publicly available database (GRUSELAMBIX; http://www.osirix-viewer.com/resources/dicom-image-library/). Selected videos depicted a series of eight views and angulations from a single case that separately included both the left and right coronary arteries; views and angulations included:

##### Left coronary artery

right anterior oblique 30˚ view (RAO30), right anterior oblique 22˚ view with 20˚ caudal angulation (RAO22/CAU20), right anterior oblique 10˚ view with 40˚ cranial angulation (RAO10/CRA40), left anterior oblique 86˚ view with 4˚ caudal angulation (LAO86/CAU4), and left anterior oblique 45˚ view with 23˚ caudal angulation (LAO45/CAU23).

##### Right coronary artery

left anterior oblique 38˚ view with 1˚ cranial angulation (LAO38/CRA1), and right anterior oblique 31˚ view with 1˚ cranial angulation (RAO31/CRA1).

##### Ventriculogram

right anterior oblique 28˚ view with 1˚ cranial angulation (RAO28/CRA1).

#### Reference diagnosis

Expert-determined (author BN) diagnosis for the case was severe single-vessel coronary artery disease (CAD; right vessel with ischaemia), with maximum diameter stenosis of 0% in the left main artery, 30% in the left anterior descending (LAD), 10% in the left circumflex artery (LCx), and 90% in the right coronary artery (RCA).

#### Eye tracker and computer

A mobile remote eye-tracking device (REDm; SMI; Boston, MA) tracked binocular eye movements at 60 Hz. The system was mounted to the bottom of a 22″ LCD computer monitor (1920 × 1080 resolution) controlled by a Samsung Series 9 laptop. Calibration was performed using the integrated SMI iView software, with a nine-point process attaining <0.5° error in visual angle.

#### Digital video viewer

To display angiogram videos, the RadiAnt DICOM Viewer (v 3.4.1; http://radiantviewer.com/) was used. The viewer allowed playback, pause, and manual frame-by-frame stepping through each of the eight videos. The SMI software captured a high-resolution screen recording of video playback and eye movement behaviour, logging all interface behaviour by tracking mouse activity (position, clicks) over time.

#### Case diagnosis report form

A six-question case report form included simple participant-specific demographics (age, experience level) and case-specific diagnostic reporting. The case-specific questions included maximum diameter stenosis for each of the four vessels (left main, LAD, LCx, and RCA), overall case-level assessment (normal, single-vessel CAD, multivessel CAD), fractional flow reserve (FFR) request (yes/no and which vessel), and overall treatment recommendation (none, pharmacotherapy, percutaneous coronary intervention/PCI, or coronary artery bypass grafting/CABG).

## Data collection procedures

Following consent, participants were provided with a basic patient medical history, including age (60 year-old male), disease (diabetes mellitus), symptoms (exertional chest discomfort), medications (aspirin, atorvastatin, metoprolol), and vital signs (blood pressure 130/90, heart rate 65 beats/min, normal electrocardiogram, normal laboratory study results). They were shown the case diagnosis report form, and then seated in front of the eye tracker and completed calibration. Participants then watched a practice coronary angiogram video and practised the interface controls. They then reviewed the series of eight angiogram videos, at their own pace and order. When they were ready, they stopped reviewing the videos and indicated their diagnostic decision on the Case Diagnosis Report Form.

## Data processing and analysis

To assess accuracy, overall percent concordance (0–100%) with expert diagnosis (across the four vessels) in reported maximum diameter stenosis was calculated. Participants were divided into two groups: *Novices *(residents) and *Experts *(attending physicians). Interface behaviour data included review time, videos viewed, and frame counts. Eye tracking data include Cartesian coordinate (*x, y *space) positions of the eyes over time, divided into fixations and saccades (and blinks removed) using published methods [43]. Outcome measures included number of fixations, mean fixation duration (in milliseconds), saccade amplitude (in degrees), and peak saccade velocity (in degrees/second).

## Statistical analyses

The nonparametric Mann-Whitney test was used to compare independent samples; the outcome of this test is the *U* statistic, which reveals moderate/marginal support (α = 0.10) or stronger support (α = 0.05) for the alternative hypothesis.

## Results

### Diagnostic accuracy

Overall diagnostic accuracy was high (*x̅* *=* 0.96, *s* *=* 0.04). Experts demonstrated higher accuracy (*x̅* *=* 1.0, *s* *=* 0) than residents (*x̅* *=* 0.95, s = 0.04); however, this difference did not reach traditional significance levels (*U* = 11.0, *p* = 0.086).

Regarding case-level assessment, FFR request, and treatment recommendation, novices and experts showed similar responses. Seven of the eight participants assessed the case as single-vessel CAD, with one novice selecting multivessel CAD. Seven of the eight participants suggested they would not request FFR, with one novice suggesting they would. Finally, seven of the eight participants recommended PCI treatment, with one novice suggesting medical therapy only. Thus, in this small sample, overall accuracy was high, though experts showed significantly higher accuracy than novices when estimating maximum diameter stenosis, and novices showed some variation in overall case assessment and treatment recommendation.

### Case review behaviour: overall

**Fig. 1:**
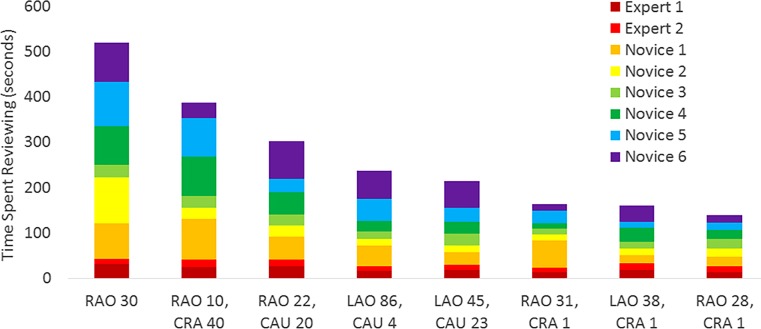
Mean time spent viewing (in seconds) each video angulation, as a function of the eight participants (2 experts, 6 novices)

Experts spent significantly less time reviewing the entire case (total review time; *x̅* *=* 132.4s; *s* *=* 41.4) relative to novices (*x̅* *=* 309.8s, *s* *=* 91.7), as depicted in Fig. 1; this difference reached significance (*U* < 0.01, *p* = 0.046). Experts also paused the video to examine individual frames less frequently (*x̅* *=* 3.0, *s* *=* 2.8) than novices (*x̅* *=* 45.5, *s* *=* 75.9), though this pattern did not reach significance (*U* = 2.5, *p* = 0.24).

### Case review behaviour: frame-specific

**Fig. 2:**
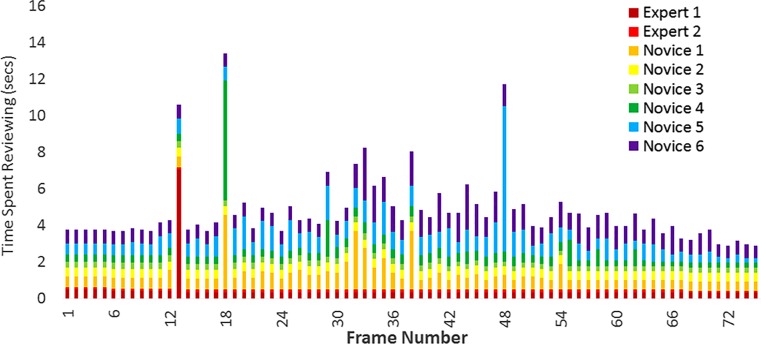
Mean time spent viewing (in seconds) each frame of RAO30, as a function of the eight participants (2 experts, 6 novices)

Frame-by-frame viewing behaviour was assessed for each video to identify frames with consistently high viewing frequency. An example frame-by-frame assessment is included in Fig. 2, for video RAO30 of the left coronary artery. To account for certain frames being increased due to a single participant’s behaviour, a z-score threshold (*x̅* + 2SD) was used for identifying critical frames. This process resulted in six critical frames distributed across five videos (RAO30, RAO22/CAU20, RAO10/CRA40, LAO45/CAU23, and LAO38/CRA1; note that RAO10/CRA40 contained two critical frames). These videos and frames were carried forward into the analysis of frame-specific eye movement behaviour.

### Eye fixations: overall

As expected given the shorter review times, experts had fewer fixations (*x̅* *=* 691, *s* *=* 352.1) than novices (*x̅* *=* 1,358.8, *s* *=* 512.6); however, when considered relative to review time, experts and novices had a similar number of fixations per second (*x̅* *=* 5.1, *s* *=* 1.08; *x̅* *=* 4.3, *s* *=* 0.55, respectively; *U* = 10, *p* = 0.18). The mean duration of fixations did not differ between experts and novices (*U* = 5, *p* = 0.74). For saccades, experts showed fewer saccades (*x̅* *=* 705, *s* *=* 370.5) than novices (*x̅* *=* 1,389.8, *s* *=* 524.9); however, when considered relative to review time, experts and novices had a similar number of saccades per second (*x̅* *=* 5.1, *s* *=* 1.2; *x̅* *=* 4.4, *s* *=* 0.55, respectively; *U* = 10, *p* = 0.18). Finally, peak saccade velocity was significantly lower in experts (*x̅* *=* 184.5, *s* *=* 34.1) than novices (*x̅* *=* 277.9; *s* *=* 90.2), *U* < 0.01, *p* = 0.046.

### Eye movement behaviour: frames and ROIs

For each of the six critical frames identified, an expert interventional cardiologist (author BN) identified a single rectangular diagnostic region; this region of interest (ROI) was determined to be the single most important region of the frame for accurately arriving at the case diagnosis. The expert also annotated the unique diagnostic information inherent to the region.

The video and frame-specific analysis examined differences in fixation and saccade parameters within versus outside the defined ROIs, as a function of cardiologist expertise. Each video and frame are described below (see Tab. 1 for descriptive statistics).

**Table 1:** Descriptive statistics

Video	Frame	Number of expert fixations	Number of novice fixations
RAO30	18	*x̅* *=* 1.5, *s* *=* 0.71	*x̅* *=* 5.2, *s* *=* 3.1
RAO22	55	*x̅* *=* 5.5, *s* *=* 3.5	*x̅* *=* 2.3, *s* *=* 1.6
RAO10	37	*x̅* *=* 0, *s* *=* 0	*x̅* *=* 3.2, *s* *=* 2.0
	54	x̅ = 5.0, *s* *=* 4.24	*x̅* *=* 11, *s* *=* 12.3
LAO45	27	*x̅* *=* 2.0, *s* *=* 2.8	*x̅* *=* 14.8, *s* *=* 19.7
LAO38	27	*x̅* *=* 2.0, *s* *=* 7.8	*x̅* *=* 7.8, *s* *=* 5.3

#### RAO30

**Fig. 3:**
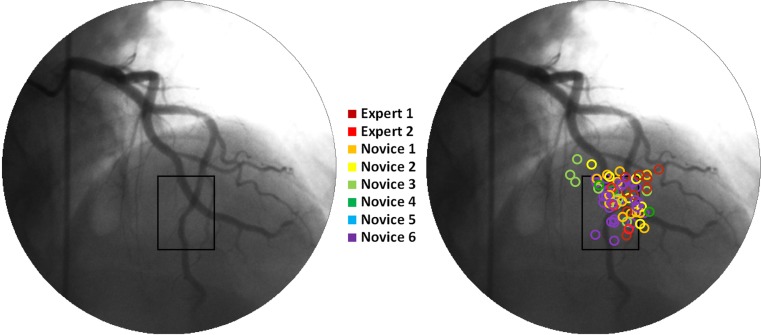
Sample critical frame derived from the ShotRank methodology, showing frame 54 of RAO10, with a critical ROI depicted as a black rectangle. Right panel shows all frame 54 fixations overlaid, color coded by participant number

This video included a single critical frame (frame 18; Fig. 3) and one expert-defined ROI. This region was annotated as follows: *This ROI shows minor disease in the ostia (i.e., opening of a small obtuse marginal branch). It also generally rules out significant disease in a major vessel (i.e., left main coronary artery). *Experts showed marginally fewer fixations on frame 18 in comparison to novices (*U* = 0.5, *p* = 0.06). Neither group, however, fixated in the ROI.

#### RAO22

This video included a single critical frame (frame 55) with one expert-defined ROI. This region was annotated as follows: *This image shows the distal LCx well and rules out significant disease in a major vessel (i.e. left main coronary artery).* Experts showed a similar number of fixations on frame 55 in comparison with novices (*U* = 9.5, *p* = 0.24). The experts showed marginally more fixations on the critical ROI (*x̅* *=* 4.0, *s* *=* 1.4) relative to novices (*x̅* *=* 1.8, *s* *=* 1.2) (*U* = 11, *p* = 0.086).

#### RAO10

This video included two critical frames, 37 and 54. On frame 37, the expert-defined ROI was annotated as follows: *Shows normal left main and proximal LAD. These are important vessels and identifying them as normal is key.* Experts showed significantly fewer fixations on frame 37 in comparison with novices (*U* = 0, *p* = 0.032). The number of fixations on the ROI did not differ between the two groups (experts *x̅* *=* 0; novices *x̅* *=* 1.5).

On frame 54, the expert-defined ROI was annotated as follows: *This shows an intermediate lesion (30% stenosis) in the mid-LAD. It is important to carefully evaluate this blood vessel. *Experts and novices showed a similar number of fixations on frame 54 (*U* = 4.0, *p* = 0.51). The number of fixations on the ROI did not differ between the two groups (experts *x̅* *=* 3.0; novices *x̅* *=* 7.3).

#### LAO45

This video included one critical frame (frame 27), with one expert-defined ROI. This region was annotated as follows: *Shows the left main and proximal LAD and LCx to be normal with some mild disease (10% stenosis) in the proximal LCx.* Experts showed significantly fewer fixations on frame 27 in comparison with novices (*U* = 0, *p* = 0.043). The experts also showed significantly fewer fixations on the critical ROI (*x̅* *=* 2.0, *s* *=* 2.8) relative to novices (*x̅* *=* 12.0, *s* *=* 15.2) (*U* = 0, *p* = 0.043).

#### LAO38

This video included one critical frame (frame 27), with one expert-defined ROI. This region was annotated as follows: *Stenosis (90%) in the mid-to-distal RCA that helps guide the need for treatment (stenting preferred in this case).* Experts and novices showed a similar number of fixations on frame 27 (*U* = 2.0, *p* = 0.18). The number of fixations on the ROI also did not differ between the two groups (experts *x̅* *=* 2.0; novices *x̅* *=* 7.7).

## Discussion

Novices showed a greater number of eye fixations and saccades, along with substantially longer review times relative to experts. They also tended to pause more to review specific frames of videos. Thus, novices display longer and more in-depth viewing relative to experts, though when diagnostic decisions were ultimately made, they did not show higher accuracy. This complements and extends similar results found with radiologists and pathologists viewing static images [2, 4, 16, 32, 44, 45]. Using a video summarization technique derived from multimedia informatics, there was evidence that novices not only fixate more on critical frames than experts, but they also consistently (with one exception, RAO30) fixate on each frame’s critical region. In most cases, they fixated the critical region with similar frequency to experts.

Results demonstrate that both experts and novices were fixating on critical frames and regions, suggesting a successful visual search process for both groups that detected and possibly recognized critical features. Second, it demonstrates that any diagnostic decision errors on behalf of novice cardiologists are not entirely driven by a failure to find and view critical features and regions of diagnostic importance, but rather to appropriately interpret them and classify the features into a cohesive diagnostic decision. For instance, per expert (author BN) annotations, the LAO45 view depicts a 10% stenosis in the left circumflex artery (LCx). Both novices and experts directly fixated on the region critical for recognizing this feature, with novices fixating it more frequently than experts; however, 5 of the 6 novices failed to correctly interpret it for diagnosis. From an educational perspective, this result suggests that trainees may need additional experience and formative feedback associating perceived visual features with diagnostic criteria; for instance, through guided discovery with feedback [46], reflection exercises [47], and interactive educational workshops with expert involvement [48].

This pilot study was the first of its kind and therefore carries some limitations. There was a small sample size that reduces statistical power and may hinder interpretation and the generalizability of any claims. For instance, while diagnostic accuracy was numerically higher in experts versus novices, this comparison did not reach statistical significance; higher power to detect differences may help elucidate this pattern. Furthermore, larger sample sizes from more diverse clinical settings would allow us to examine whether predictive relationships exist between eye movements and diagnostic outcomes, assessed through regression-based analyses. Given the preliminary nature of this study, concrete recommendations for education and training are not possible; however, the results provide some suggestion that eye tracking holds potential for assessing expertise development in specialized medical domains and providing objective formative feedback to students [26].

By better discerning different eye movement patterns across medical professionals, physicians, scientists, educators, and practitioners may be opening up the ‘black box’ of visual behaviour that has been previously described as being both unreliable and inaccurate [49–52]. For instance, in one study, investigators identified that individual cardiologists’ interpretations of angiograms in New York was frequently at odds with the interpretation by expert panels and this led to substantial differences in referral for major interventional and surgical procedures [53]. In addition, these findings could form the framework for developing automated systems of visual interpretation that assist cardiologists through algorithms that employ signal processing and machine learning techniques that mimic the actual visual behaviour of experts.

## Conclusions

In conclusion, this study supports the feasibility of tracking and interpreting eye movements during medical video interpretation, and demonstrates expertise-related differences in interpretive behaviour. Novices and experts alike fixate critical video segments and regions, but novices show some interpretive failures that lead to under- or over-diagnosis. This finding supports and extends research done with medical domains using static images, such as radiology and pathology, and provides a more nuanced understanding of how decision errors may arise when physicians interpret medical images. Continuing research will need to explore these issues in depth and examine their relevance for guiding training and evaluation of physicians.

## Abbreviations

CABG: coronary artery bypass grafting
CAD: coronary artery disease
CAU: caudal
CRA: cranial
DICOM: digital imaging and communications in medicine
FFR: fractional flow reserve
LAD: left anterior descending
LAO: left anterior oblique
LCD: liquid crystal display
LCx: left circumflex artery
PCI: percutaneous coronary intervention
RAO: right anterior oblique
RCA: right coronary artery
REDm: remote eye-tracking device mobile
ROI: region of interest
SMI: SensoMotoric Instruments
